# Functional development of photoreceptors in human retinal organoids

**DOI:** 10.1186/s13287-026-05027-z

**Published:** 2026-04-30

**Authors:** Yue Zhang, Mingxia Du, Yi-Han Wang, Min Li, Kangxin Jin, Deng Pan, Xiao Zhang, Zi-Bing Jin

**Affiliations:** 1 Beijing Institute of Ophthalmology, Department of Ophthalmology, Beijing Tongren Eye Center, Beijing Tongren Hospital, Capital Medical University, Beijing Key Laboratory for Ophthalmic Cell and Gene Therapy, Beijing, 100008 China; 2 Laboratory for Clinical Medicine, Capital Medical University, Beijing, 100069 China

**Keywords:** Retinal organoids, hESC, Whole-cell patch-clamp, Photoreceptor, Ion channel

## Abstract

**Background:**

Retinal organoids (ROs) derived from human pluripotent stem cells are crucial for modeling retinal development and disease. However, the functional electrophysiological maturation of photoreceptors within ROs remains poorly characterized. This study aimed to define the functional maturation timeline of photoreceptors in human embryonic stem cell (hESC)-derived ROs.

**Methods:**

H9 hESC-derived ROs which included a CRX-tdTomato reporter line for specific photoreceptor identification were utilized. An integrated approach of RNA-sequencing analysis, immunofluorescence staining, and whole-cell patch-clamp recordings was employed to systematically assess photoreceptor maturation over 300 days of differentiation.

**Results:**

Transcriptional and protein analysis revealed progressive upregulation of key ion channels. Patch-clamp recordings demonstrated stage-dependent maturation of membrane properties, which stabilized by D120–125. Hyperpolarization-activated cyclic nucleotide-gated (HCN) channel-mediated currents (I_*h*_) increased progressively, peaking at D240, with amplitudes comparable to mature primate photoreceptors. Voltage-gated sodium (Nav) currents also showed significant developmental upregulation, reaching a maximum, stable plateau from D210–215 onward. Pharmacological blockade confirmed the identity of HCN and Nav currents. Critically, the capacity for action potential (AP) generation increased developmentally, with the proportion of photoreceptors capable of firing APs rising from 16.7% at D90–95 to a peak of 90.2% by D240–245.

**Conclusions:**

This study defines a comprehensive electrophysiological maturation timeline for photoreceptors in human ROs and establishes D240 as a key benchmark for functional maturity, characterized by peak I_*h*_ currents and AP generation capacity equivalent to mature native photoreceptors. These findings provide essential physiological criteria for standardizing RO quality control, enhancing their utility for modeling retinal degenerative diseases and developing cell replacement therapies.

**Supplementary Information:**

The online version contains supplementary material available at10.1186/s13287-026-05027-z.

## Introduction

Retinal degenerative diseases, including age-related macular degeneration (AMD) and retinitis pigmentosa (RP), are among the primary contributors to irreversible vision loss globally [1–3]. A hallmark of these diseases is the progressive dysfunction or loss of photoreceptors, which critically impairs the fundamental phototransduction and synaptic transmission processes, representing the primary cause of blindness in AMD and RP [4–6]. Current treatments are limited, primarily focusing on slowing disease progression rather than restoring lost vision [7, 8]. Stem cell‑derived photoreceptors offer a promising avenue for retinal repair [9]; yet their therapeutic potential hinges critically on the ability to exhibit native electrophysiological functionality [10, 11].

Pluripotent stem cell-derived retinal organoids (ROs) provide powerful in vitro models for studying retinal development and disease, recapitulating the 3D structure, cellular diversity, and developmental progression of the human retina [11–15]. Single-cell transcriptomics confirms their trajectory closely mirrors native retina development, with transcriptomes stabilizing and converging with mature peripheral retinal cell types around 30–38 weeks [16]. However, transcriptomic similarity does not equate to functional maturity. Neuronal integration critically depends on ion channel expression and electrophysiological network formation [17–19]. Notably, while markers like recoverin are expressed early in differentiation and rhodopsin emerges at mid-stage [20, 21], the developmental phase defining functionally mature photoreceptors remains unresolved. This underscores the necessity of staged electrophysiological assessment to evaluate functional maturity in ROs.

Photoreceptors’ functional integrity fundamentally relies on the expression and coordinated activity of key ion channels [22, 23]. Hyperpolarization-activated cyclic nucleotide-gated (HCN) channels stabilize the resting membrane potential and enable post-hyperpolarization recover via the inward *h* current (I_*h*_), while voltage-gated sodium (Nav) channels contribute to action potential initiation and propagation at synaptic terminals [24–26]; additionally, cyclic nucleotide-gated (CNG), voltage-gated potassium (Kv), calcium-activated chloride (Cl^−^), and voltage-gated calcium (Cav) channels collectively regulate photoreceptor excitability and signaling [27–29]. Therefore, elucidating the developmental electrophysiological properties of these channels within RO photoreceptors is essential for assessing functional integrity, validating ROs as human retinal disease models, and establishing their transplantation potential. Despite previous studies using patch-clamp and multielectrode array techniques have provided initial insights into the electrophysiological characteristics of ROs [10, 30], a detailed analysis of the ion channel expression profiles in photoreceptors across distinct developmental stages remains lacking.

In order to identify photoreceptors more accurately, we utilized CRX-tdTomato reporter H9 embryonic stem cell (ESC)-derived ROs in which the photoreceptors were labeled with red fluorescent protein tdTomato [31]. By integrating RNA-seq, immunofluorescence staining, and whole-cell patch-clamp recordings, we established a systematic developmental profile of human RO photoreceptor electrophysiology over 300 days. Our analysis revealed progressive maturation of HCN and Nav channel-mediated currents and action potential generation capacity during differentiation, with D240 emerging as the peak of functional maturation where electrophysiological properties comparable to those reported in mature primate and human photoreceptors. These findings not only provide a validated physiological timeline for RO differentiation but also establish functional benchmarks for modeling retinal diseases.

## Materials and methods

### Human ESC maintenance and generation of 3D retinal organoids

The hESC lines used in this study were the H9 line (WiCell, WA09; hPSCReg ID, WAe009-A) and a genetically modified H9-CRX-tdTomato line, generated in-house as previously reported [31]. Undifferentiated hESCs were maintained on vitronectin (rp01002, Shownin) in E8 medium (05990, STEMCELL) and passaged every 4–5 days using 0.5 mM EDTA (15575020, Gibco) at split ratios of 1:30 to 1:40. Cells were cryopreserved using a high-efficiency freezing medium (SN-06-1210, Shownin) and consistently tested negative for mycoplasma contamination using the Mycoplasma Detection Kit (CA6101024, CELLAPY bio; Figure S1A). Short tandem repeat (STR) profiling was performed to authenticate cell line identity (Figures S1B and S1C). All experiments were conducted with cells between passages 40 and 60.

RO differentiation was performed based on previously described protocols with modifications [32]. Briefly, hESC colonies were rinsed with DPBS (C14190500BT, Gibco), treated with dispase (1 mg/mL, 07923, STEMCELL) for 3–4 min until colony edges lifted, and washed with DMEM/F12 medium (C11330500BT, Gibco). Colonies were manually fragmented into 6–9 pieces (per one colony) using a 10 µL pipette tip, harvested by centrifugation (200 ×g, 5 min), and resuspended in ice-cold Matrigel (3536-005-02, R&D) for 20 min at 37 °C. Cell aggregates were cultured in N2B27 medium consisting of DMEM/F12 : Neurobasal medium (21103049, Gibco) (1:1), 0.5× B27 supplement (17504044, Gibco), 0.5× N2 supplement (17502048, Gibco), 0.1 mM β-mercaptoethanol (M3148, Sigma-Aldrich), and 0.2 mM L-GlutaMAX (35050061, Gibco) under 5% CO₂ at 37 °C (designated day 0). Adherent colonies formed by day 7 were treated with dispase from day 13–15 to induce detachment and then cultured in B27 medium containing DMEM (C11965500BT, Gibco): F12 (C11765500BT, Gibco) (3:1), 2% B27, and 1× NEAA (M7145, Sigma-Aldrich). Half-medium changes were performed every 3 days using a transfer pipette. One week post-detachment, cultures were transitioned to serum medium containing DMEM: F12 (3:1), 2% B27, 1× NEAA, 8% fetal bovine serum (C04002-500, VivaCell), 100 mM taurine (T0625, Sigma-Aldrich), and 2 mM GlutaMAX, with weekly medium replacement. ROs were harvested at designated stages for immunostaining and electrophysiological studies.

### RNA sequencing

Publicly available RNA-seq data (accession: GSE136929) from the Gene Expression Omnibus (GEO) database were reanalyzed to investigate the expression profiles of key photoreceptor-related genes [13].

### Immunofluorescence staining

Following fixation in 4% paraformaldehyde (30 min, room temperature), ROs were washed with DPBS, embedded in OCT compound (4583, Sakura Finetek), and rapidly frozen. 10-µm thick sections were cut using a cryostat microtome. For staining, the sections were firstly washed in PBS (3 × 10 min), blocked with 4% bovine serum albumin (BSA) in PBS containing 0.5% Triton X-100 for 1 h at room temperature, then incubated overnight at 4 °C with primary antibodies diluted in blocking solution. On the next day, sections were washed in PBS (3 × 10 min) and incubated with species-specific fluorophore-conjugated secondary antibody diluted in blocking solution containing 0.1% DAPI (D1306, Thermo Fisher) for 1 h in the dark at room temperature. After being washed in PBS (3 × 10 min), the sections were mounted using anti-fade mounting media (345789, Millipore). Imaging was performed using Olympus SPIN SR laser scanning confocal microscope. Primary antibodies used in the study included: mouse anti-HCN1 (75–110, NeuroMab, 1:100), goat anti-NRL (AF2945, R&D, 1:100), rabbit anti-Opsin Red/Green (MAB5405, Millipore, 1:100), rabbit anti-Pan-Na (ASC-003, alomone lab, 1:100), mouse anti-RxRγ (sc-365252, Santa Cruz, 1:200), mouse anti-rhodopsin (MAB5316, Millipore, 1:100). Secondary antibodies used in the study included: donkey anti-mouse Alexa Fluor 488 (A21202, Invitrogen, 1:500), donkey anti-goat Alexa Fluor 647 (A32849, Invitrogen, 1:500), donkey anti-rabbit Alexa Fluor 647 (A31573, Invitrogen, 1:500).

### Whole-cell patch-clamp recording

The 3D-cultured ROs were carefully moved to the recording chamber on an upright microscope (Olympus BX51WI, Japan) and continuously perfused with Ames’ solution containing (in mM) 120 NaCl, 3.1 KCl, 1.1 CaCl_2_, 1.2 MgSO_4_, 0.5 KH_2_PO_4_, 22.6 NaHCO_3_, 6 D-glucose, and 0.5 l-glutamine bubbled with 95% O_2_ and 5% CO_2_ (pH 7.4) at a rate of 2 mL/min. The experimental bath temperature was regulated between 30 and 32 °C using a heater controller (TC-344 C, Warner Instruments, USA). Following visual identification of tdTomato-expressing neurons under fluorescence microscopy, whole-cell recordings were performed in either current- or voltage-clamp mode. The electrophysiological measurements were acquired using an Axopatch 200B amplifier and a Digidata 1550B digitizer (Molecular Devices, USA). Patch pipettes were pulled from borosilicate glass capillaries (Sutter Instruments, USA, OD 1.5 mm, ID 0.86 mm) using a Sutter P-1000 puller (Sutter Instruments, USA) and filled with a K^+^-based internal solution containing (in mM) 110 KCl, 13 NaCl, 1 CaCl_2_, 2 MgCl_2_, 10 EGTA, 5 MgATP, 0.5 NaGTP and 10 HEPES, adjusted to pH 7.2 with KOH. Internal solution osmolarity was adjusted to 280–290 mOsm/L, and electrode resistance was 4–9 MΩ in bath solution. Data were acquired using a pClamp10 system (Molecular Devices, USA), low-pass filtered at 2 kHz and sampled at 10 kHz. The value of the liquid junction potential in this study has not been corrected.

The specific sources and catalog numbers for all chemicals and pharmacological agents used are as follows: NaCl (S9888, Sigma-Aldrich), KCl (P5405, Sigma-Aldrich), CaCl_2_ (C7902, Sigma-Aldrich), MgSO_4_ (M1880, Sigma-Aldrich), KH_2_PO4 (P5655, Sigma-Aldrich), NaHCO_3_ (S6014, Sigma-Aldrich), d-glucose (G8270, Sigma-Aldrich), l-glutamine (G8540, Sigma-Aldrich), MgCl_2_ (M2670, Sigma-Aldrich), EGTA (E0396, Sigma-Aldrich), MgATP (A9187, Sigma-Aldrich), NaGTP (G8877, Sigma-Aldrich), HEPES (H4034, Sigma-Aldrich), CsCl (C4036, Sigma-Aldrich), ZD7288 (Z3777, Sigma-Aldrich), BaCl_2_ (342920, Sigma-Aldrich), and TTX (B-CHM431, BiogradeTech). TTX, CsCl, ZD7288, and BaCl_2_ were prepared as stock solutions in sterile water (1000× final concentration), aliquoted, and stored at − 20 °C until dilution in Ames’ solution was required.

### Data analysis

All data were obtained from a total of at least five ROs derived from a minimum of three independent differentiations. Quantification of immunofluorescence signal intensity was performed on confocal images using ImageJ (National Institutes of Health, USA). For each marker (HCN1, Pan-Na), consistent regions of interest (ROIs) encompassing the photoreceptor layer were defined. The mean fluorescence intensity per ROI was measured, followed by background subtraction using adjacent non-specific areas. Intensity values for each time point (D150, D240, D310) were then normalized to the mean background-corrected intensity of the D90 group. Electrophysiological data were analyzed offline using MATLAB (MathWorks, USA) and Clampfit 10.7 (Molecular Devices, USA). All data were presented as mean ± SEM. Statistical analyses were conducted using GraphPad Prism 9 (GraphPad Software, USA), including two-tailed paired t-tests and either one-way or two-way ANOVA with Tukey’s post hoc comparisons. Statistical significance was determined at the level of *P* < 0.05.

## Results

### RNA-seq and immunofluorescence analysis of functional maturation in hESC-derived ROs

**Fig. 1:**
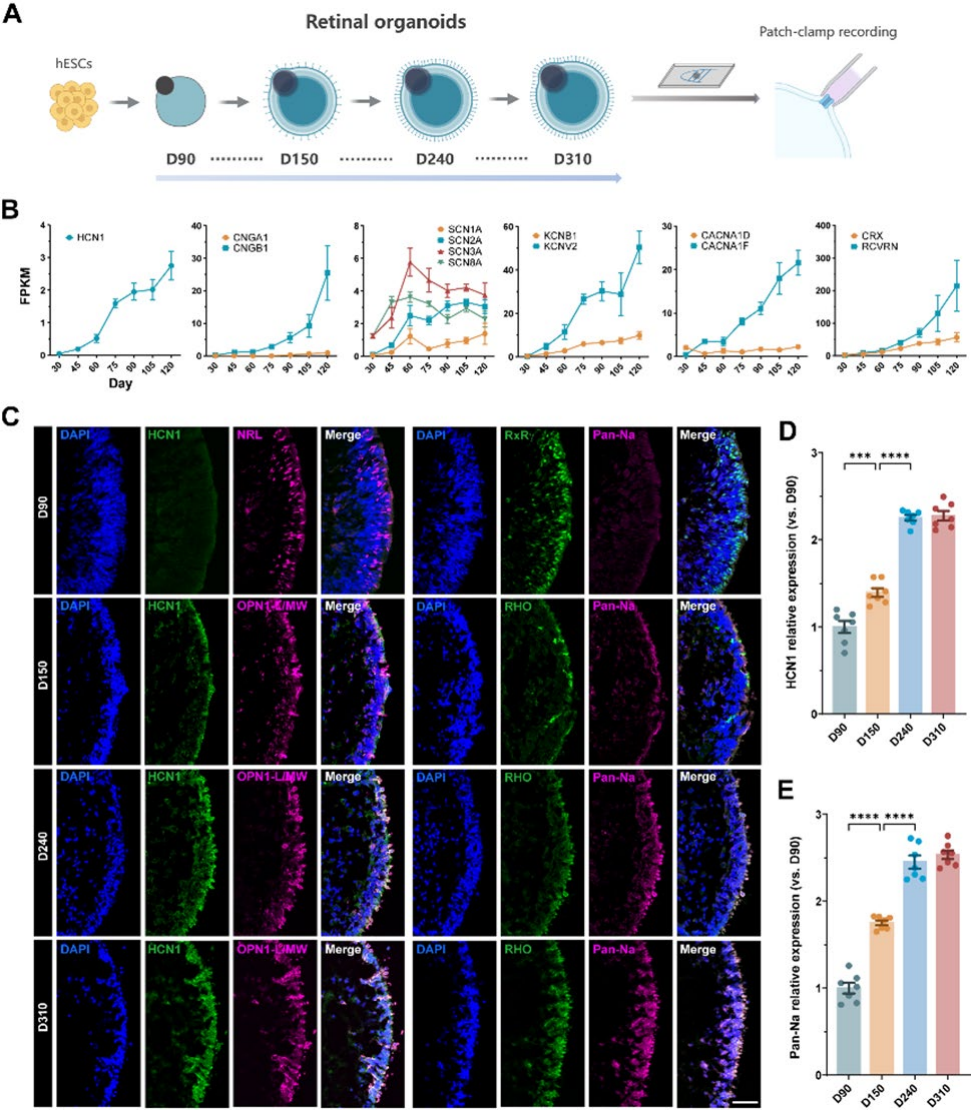
Temporal expression profiles of ion channel and marker genes in ROs across differentiation stages. **A** Schematic overview of retinal organoid differentiation and electrophysiological assessment. **B** Temporal expression patterns of key retinal ion channel genes and markers in ROs from day 30 to day 120 as assessed by RNA-seq. Data were presented as mean FPKM (Fragments Per Kilobase Million) values ± SEM (*n* = 3 at D30/105; *n* = 4 at D120; *n* = 5 at D45/60/75/90). Expressions of HCN channel subunit *HCN1*, CNG channel subunit *CNGB1*, Nav channel subunits *SCN1A/SCN2A*, Kv channel subunit *KCNB1/KCNV2*, Cav channel subunit *CACNA1F* and photoreceptor markers recoverin (*RCVRN)/CRX* gradually increase during differentiation. Expression of CNG channel subunit *CNGA1* and Cav channel subunit *CACNA1D* remained at low levels throughout the differentiation timeline. In contrast, the expression of Nav channel subunits *SCN3A* and *SCN8A* peaked at day 60 and subsequently decreased. **C** Immunofluorescence analysis of HCN1 (green) and Nav channel expression (Pan-Na, pink) in ROs at different differentiation stages (D90, D150, D240, D310). NRL (Nuclear receptor subfamily 1), RxRγ (Retinoid X receptor gamma), OPN1-L/MW (Opsin 1, long/middle-wave sensitive cone opsin), and RHO (Rhodopsin) are key photoreceptor markers expressed during both early and mature stages of differentiation. Nuclei were stained with DAPI (blue). Scale bar = 50 μm. **D**, **E** Quantification of HCN1 (**D**) and Pan-Na (**E**) expression. Data were presented as mean ± SEM (*n* = 7 per group), and statistical significance was determined using one-way ANOVA with Tukey’s multiple comparisons test, ***P < 0.0001, ****P < 0.0001

Our study utilized H9 hESC-derived ROs as a model system to dissect photoreceptor functional maturation across a prolonged differentiation timeline (Fig. 1A). We first performed transcriptional analysis using existing RNA-seq data (GEO: GSE136929) [13]. Time-course analysis across seven differentiation stages (D30–120) revealed sequential upregulation of ion channel transcripts critical for photoreceptor electrophysiology (Fig. 1B). In contrast to the general trend, analysis of Nav channels revealed a complex transcriptional profile: *SCN2A* expression increased progressively, whereas *SCN3A* and *SCN8A* expression peaked at D60 and subsequently decreased. Conversely, HCN subunit *HCN1*, CNG subunit *CNGB1*, as well as Kv channels (*KCNB1*, *KCNV2*) and Cav channels (*CACNA1F*) all exhibited a pattern of gradual transcriptional upregulation from D30 through D120. This expression pattern paralleled the acquisition of photoreceptor-specific markers, recoverin (*RCVRN*, a calcium sensor regulating phototransduction recovery), and the transcription factor cone-rod homeobox (*CRX*, a master regulator of photoreceptor development), suggesting coordinated molecular maturation toward functional photoreceptors.

To validate the transcriptional dynamics at the protein level, we next examined the protein expression of HCN and Nav channels across prolonged differentiation (D90–310). Immunofluorescence analysis revealed stage-dependent accumulation of HCN1 and pan-Na signals (Fig. 1C), with quantitative intensity measurements demonstrating progressive increases from D90 to D310 (Fig. 1D and E). Notably, HCN1 exhibited a maturation-dependent activation pattern: barely detectable at D90, significantly upregulated by D150, and peaking at D240 (Fig. 1D). Similarly, pan-Na expression remained low at D90 but showed marked elevation from D150, sustaining high levels through D310 (Fig. 1E). These results were consistent with the RNA-seq data, demonstrating a progressive increase in the expression of HCN and Nav channels at both the transcriptional and protein levels, reflecting the functional maturation of ROs during differentiation.

### Progressive maturation of electrophysiological properties of RO photoreceptors

**Fig. 2:**
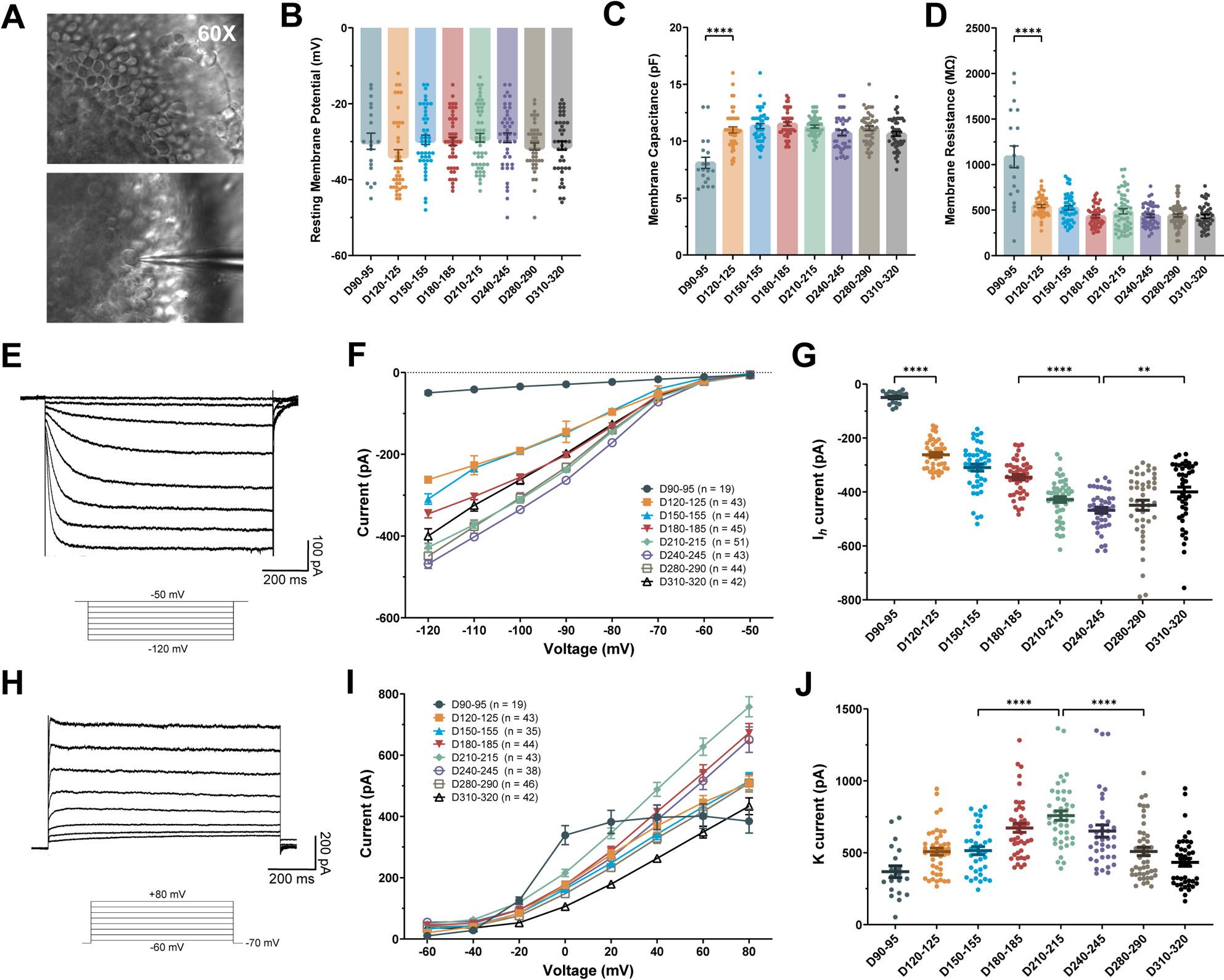
Electrophysiological maturation of RO photoreceptors across different differentiation stages. **A** Representative images of the whole-cell patch-clamp recordings applied to RO photoreceptors to assess their electrophysiological properties. **B**–**D** Intrinsic membrane properties of RO photoreceptors at various stages of differentiation, including (**B**) resting membrane potential (RMP; D90–95, n = 19; D120–125, n = 42; D150–155, n = 45; D180–185, n = 45; D210–215, n = 51; D240–245, n = 43; D280–290, n = 45; D310–320, n = 42), **C** membrane capacitance (Cm; D90–95, n = 19; D120–125, n = 43; D150–155, n = 45; D180–185, n = 45; D210–215, n = 51; D240–245, n = 43; D280–290, n = 45; D310–320, n = 48), and **D** membrane resistance (Rm; D90–95, n = 19; D120–125, n = 43; D150–155, n = 45; D180–185, = 45; D210–215, n = 51; D240–245, n = 43; D280–290, n = 60; D310–320, n = 42). These properties of RO photoreceptors began to mature from D120 of differentiation. **E** Representative traces of hyperpolarization- activated current (Ih current) in a RO photoreceptor (top traces), elicited by hyperpolarizing voltage steps from − 120 mV to − 50 mV in 10-mV increments, held at a potential of − 50 mV (bottom traces). **F** Current– voltage relationships of Ih current (measured at steady state) for RO photoreceptors at various different differentiation stages (n = number of cells). **G** Comparisons of Ih current amplitude (measured at steady state) at voltage of − 120 mV (D90–95, n = 19; D120–125, n = 42; D150–155, n = 45; D180–185, n = 45; D210–215, n = 51; D240–245, n = 43; D280–290, n = 44; D310–320, n = 42). The Ih current exhibited a progressive increase during RO differentiation, peaking around D240. **H** Representative potassium (**K**) current (top traces) of a RO photoreceptor in response to voltage steps from − 60 mV to + 80 mV in 20-mV increments (bottom traces), held at a potential of − 70 mV. **I** Current–voltage curve of K currents for RO photoreceptors at various different differentiation stages (n = number of cells). **J** Comparisons of K current amplitude at voltage of + 80 mV (D90–95, n = 20; D120–125, n = 43; D150–155, n = 35; D180–185, n = 44; D210–215, n = 43; D240–245, n = 38; D280–290, n = 46; D310–320, n = 42). K current demonstrated a differentiation-stage-dependent increase, culminating in peak values at D210. Data were presented as mean ± SEM, and statistical significance was determined using one-way ANOVA with Tukey’s multiple comparisons test, **P < 0.01, ****P < 0.0001

To assess the electrophysiological maturation of RO photoreceptors, we conducted whole-cell patch-clamp recordings targeted at cells based on their tdTomato fluorescence in the neural retina outer layers (Fig. 2A). The identity of these tdTomato-positive cells as photoreceptors was confirmed by co-localization with the photoreceptor markers CRX (Fig. S2A) and RCVRN (Fig. S2B). Measurements of intrinsic membrane properties revealed minimal variation in resting membrane potential (RMP) across differentiation (D90–320; Fig. 2B). In contrast, membrane capacitance (Cm) increased significantly from 8.1 ± 0.5 pF at D90–95 to 11.0 ± 0.3 pF at D120–125 (*P* < 0.0001; Fig. 2C), while membrane resistance (Rm) decreased markedly from 1085.0 ± 119.4 MΩ to 543.7 ± 17.6 MΩ over the same period (*P* < 0.0001; Fig. 2D). Critically, both Cm and Rm stabilized after D120–125 with no significant later changes, indicating completion of early structural and functional maturation by this developmental stage.

We next evaluated functional maturation of hyperpolarization-activated currents (I_*h*_), which are pivotal for photoreceptor electrophysiology. Under voltage-clamp conditions (holding potential: − 50 mV), representative I_*h*_ current traces were elicited by hyperpolarizing steps from − 120 mV to − 50 mV (Fig. 2E and Fig. S3). Steady-state current-voltage analysis revealed stage-dependent increase of I_*h*_ current amplitudes (Fig. 2F), with the − 120 mV response increasing progressively: from − 49.7 ± 5.1 pA at D90–95 to − 468.4 ± 10.9 pA at D240–245 (peak amplitude), followed by modest decline to − 399.9 ± 18.0 pA at D310–320 (Fig. 2G). This trajectory demonstrates sustained functional maturation of I_*h*_ currents, achieving maximal amplitude at D240–245 that approximates mature in vivo photoreceptor physiology. The subsequent amplitude attenuation suggests declining functional integrity in prolonged cultures.

The outward currents, which resemble the A-type K current (I_A_) commonly observed in various retinal neuronal types [33], were also examined throughout the maturation process of RO photoreceptors. Representative traces of K^+^ currents in RO photoreceptors were elicited by voltage steps from − 60 mV to + 80 mV, held at a potential of − 70 mV (Fig. 2H). Current–voltage analysis demonstrated a gradual increase in amplitude, with the maximum current observed at D210–215 (Fig. 2I and J). Specifically, the outward current amplitude at + 80 mV increased from 367.9 ± 40.5 pA at D90–95 to 758.0 ± 32.5 pA at D210–215 (peak amplitude), followed by gradual decline to 433.4 ± 27.4 pA at D310–320 (Fig. 2J). This transient developmental profile indicates stage-specific Kv channel maturation, contributing to membrane potential stabilization and excitability regulation during photoreceptor development.

### Functional characterization of HCN channels in RO photoreceptors

**Fig. 3:**
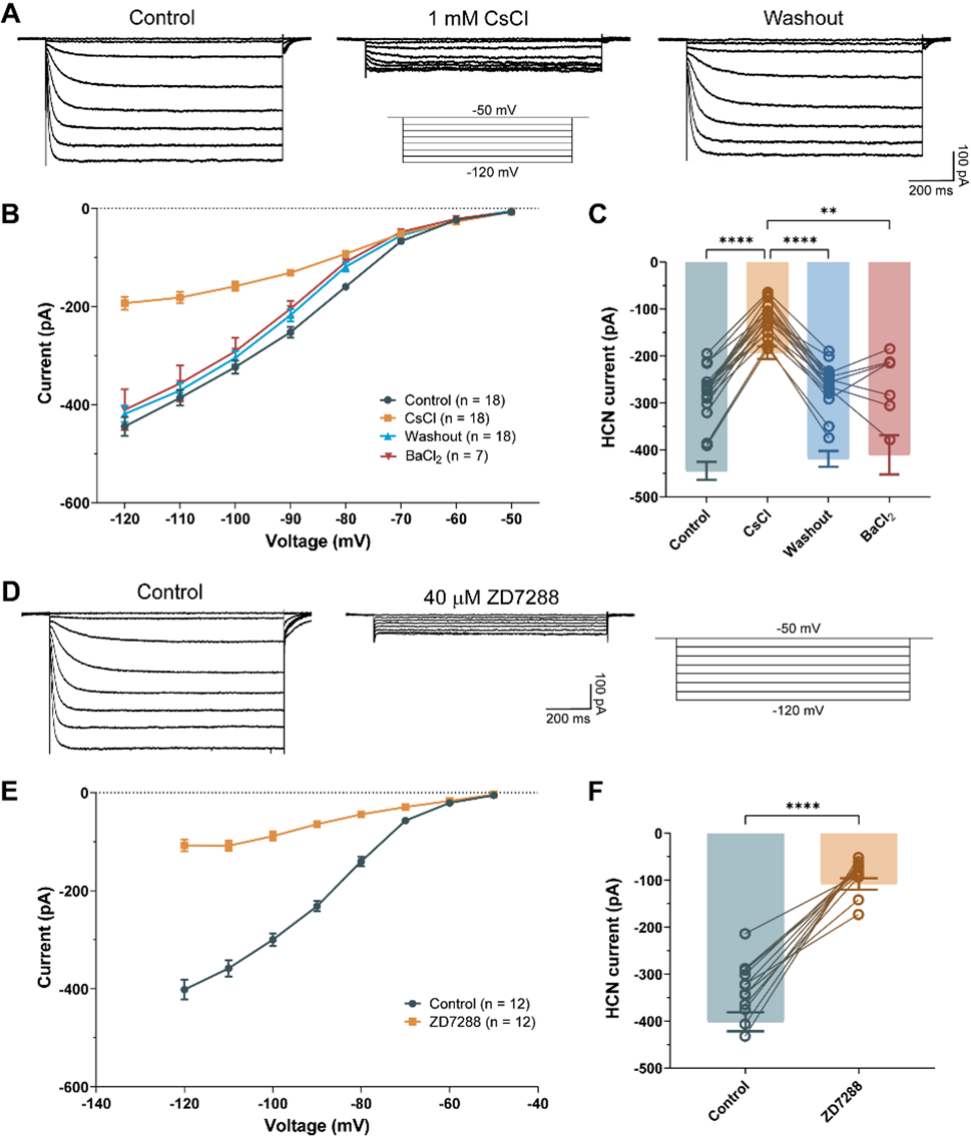
Characterization of HCN currents in RO photoreceptors. **A** Representative traces of HCN current of an individual RO photoreceptor in response to hyperpolarizing voltage steps from a holding potential of − 50 mV under control conditions, after application of 1 mM CsCl, a non-specific blocker of HCN channels, and recovery of HCN current after CsCl washout. **B** Current–voltage curve (measured at steady state) of HCN currents under control conditions (*n* = 18), following application of 1 mM CsCl (*n* = 18), after washout (*n* = 18), and following application of 200 µM BaCl_2_, an anomalous rectifier K channel blocker (*n* = 7). **C** Comparisons of plateau HCN current amplitude at − 120 mV under the respective control, drug application, and washout conditions as schematized in A. The HCN currents were partially inhibited by the application of 1 mM CsCl and subsequently recovered after washout. In contrast, the application of 200 µM BaCl_2_ had no effect on the HCN current (Control, *n* = 18; CsCl, *n* = 18; Washout, *n* = 18; BaCl_2_, *n* = 7). One-way ANOVA with Tukey’s multiple comparisons test, **P < 0.01, ****P < 0.0001. **D** Representative traces of HCN currents of an individual RO photoreceptor in response to hyperpolarizing voltage steps from a holding potential of − 50 mV before and after application of 40 µM ZD7288, a specific HCN channel blocker. **E** Current–voltage curve (measured at steady state) of HCN currents under control conditions (*n* = 12) and after application of 40 µM ZD7288 (*n* = 12). **F** Comparisons of plateau HCN current amplitude at − 120 mV under the conditions described in D. The HCN currents were mostly suppressed by the application of 40 µM ZD7288 (*n* = 12, two-tailed paired t test, ****P < 0.0001)

For these electrophysiological experiments, ROs at day 240–245 of differentiation were used. Under voltage-clamp recordings, we found that RO photoreceptors exhibited substantial inward currents (I_*h*_ current) at hyperpolarized voltage steps (Fig. 2E and G), closely resembling those observed in macaque foveal cones [10]. Previous research has attributed these large I_*h*_ currents to HCN ion channels, which are commonly found in vertebrate cones and play a crucial role in shaping the gain and kinetics of cone photovoltages [34, 35]. To investigate the presence of HCN channels in RO photoreceptors, we performed a series of voltage-clamp recordings under various experimental conditions (Fig. 3A). Under control conditions, RO photoreceptors exhibited large inward I_*h*_ currents characteristic of HCN channels. Bath application of 1 mM CsCl (3–5 min), a non-specific HCN channel blocker, significantly reduced these currents, confirming the involvement of HCN channels. This inhibition was reversible, as the currents recovered following a ≥ 10-minute CsCl washout with drug-free Ames’ solution. In contrast, application of 200 µM BaCl_2_ (3–5 min), a blocker of anomalous rectifier K channels, had no significant effect on the HCN currents (Fig. 3B and C). The plateau HCN current amplitude at − 120 mV was − 444.4 ± 19.1 pA under control conditions, which decreased to − 193.1 ± 13.3 pA after CsCl application and recovered to − 419.1 ± 16.9 pA after CsCl washout (Fig. 3C). Whereas application of 200 µM BaCl_2_ resulted in a current amplitude of − 410.6 ± 41.7 pA, indicating no significant effect on HCN currents (Fig. 3C). To further confirm the presence of HCN channels, we repeated this experiment by bath application of 40 µM ZD7288 (3–5 min), a specific HCN channel blocker, which almost completely eliminated the inward I_*h*_ currents in RO photoreceptors (Fig. 3D and F). The plateau HCN current amplitude at − 120 mV was − 401.6 ± 20.2 pA under control conditions, which decreased to − 107.7 ± 12.1 pA after bath application of ZD7288 (Fig. 3F). Notably, the inhibition by ZD7288 was not reversible within the timeframe of our washout protocol (≥ 10 min), consistent with its known high-affinity and often irreversible binding to HCN channels. This differential reversibility, where currents recovered after CsCl washout but not after ZD7288 application, further validates the pharmacological identity of the currents and aligns with the distinct mechanisms of action of these two blockers. This finding confirms the substantial contribution of HCN channels to the observed currents.

### Progressive maturation of Nav channels in RO photoreceptors

**Fig. 4:**
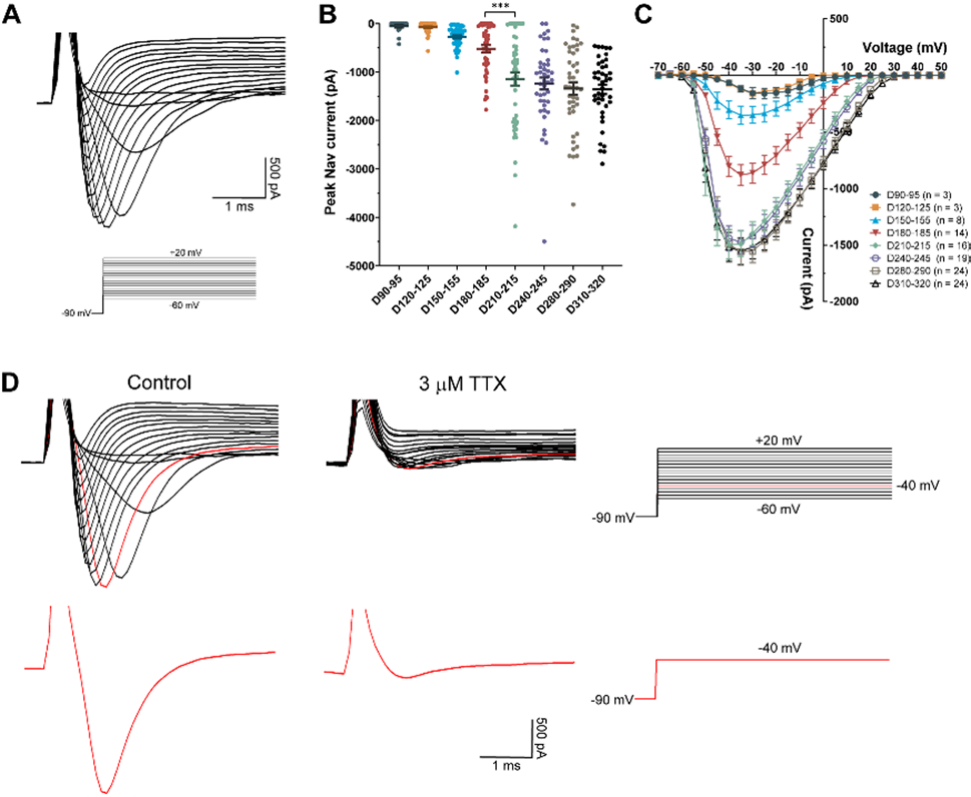
Characterization of Voltage-gated sodium currents in RO photoreceptors. **A** Representative sodium (Nav) current (top traces) elicited by voltage steps from − 60 mV to + 20 mV in 5-mV increments at holding potential of − 90 mV (bottom traces). **B** Comparison of peak Nav current amplitudes among photoreceptors at different stages of differentiation. The n values indicate the total number of photoreceptors recorded for each stage (D90–95, *n* = 19; D120–125, *n* = 43; D150–155, *n* = 44; D180–185, *n* = 45; D210–215, *n* = 50; D240–245, *n* = 43; D280–290, *n* = 44; D310–320, *n* = 42). This analysis includes all recorded cells, regardless of whether a measurable current was present. Data were presented as mean ± SEM, and statistical significance was determined using one-way ANOVA with Tukey’s multiple comparisons test, ***P < 0.001. The Nav current amplitude increased progressively during differentiation, reaching a maximum level by D210–215 and sustaining this plateau through the latest stage examined (D310–320). **C** Current-voltage relationship of Nav currents at corresponding stages, analyzed from a subset of photoreceptors that exhibited a measurable Nav current (> 100 pA) with a stereotypical waveform and minimal noise (n = number of cells). Data were presented as mean ± SEM. The curves were fitted using Boltzmann function to determine the voltage dependence of sodium channel activation. **D** Representative traces of Nav current of an individual RO photoreceptor in response to voltage steps from − 60 mV to + 20 mV at holding potential of − 90 mV under control conditions and after application of 3 µM tetrodotoxin (TTX), a voltage-gated sodium channel blocker. Lower panels illustrate the peak Nav current from the same cell as the upper panels in response to voltage steps at − 40 mV. Right traces, voltage waveform used to elicit Nav currents. The Nav current was blocked by 3 µM TTX

To characterize the functional development of Nav channels in RO photoreceptors, we performed voltage-clamp recordings. Representative Nav current traces were elicited by voltage steps from − 60 mV to + 20 mV at a holding potential of − 90 mV (Fig. 4A and Fig. S4). Developmental analysis demonstrated a progressive amplification of peak Nav current amplitudes (Fig. 4B and C). Currents initiated from minimal levels at D90–95 (− 53.1 ± 27.3 pA), increased progressively, and underwent a significant maturation step between D180–185 and D210–215, reaching a stable, maximal level by D210–215 (− 1144.0 ± 136.5 pA). This mature current amplitude was then maintained without further significant increase through D310–320. Next, to confirm the identity of the Nav currents, we performed pharmacological analyses. Bath application of 3 µM TTX (3–5 min), a selective Nav channel blocker, effectively abolished these currents in RO photoreceptors (Fig. 4D), indicating that the observed currents are mediated by Nav channels. Collectively, this progressive and sustained enhancement, reaching a stable functional plateau from D210–215 onward, demonstrates continuous maturation of Nav channels throughout photoreceptor development.

### Electrophysiological characterization of membrane potentials in RO photoreceptors

**Fig. 5:**
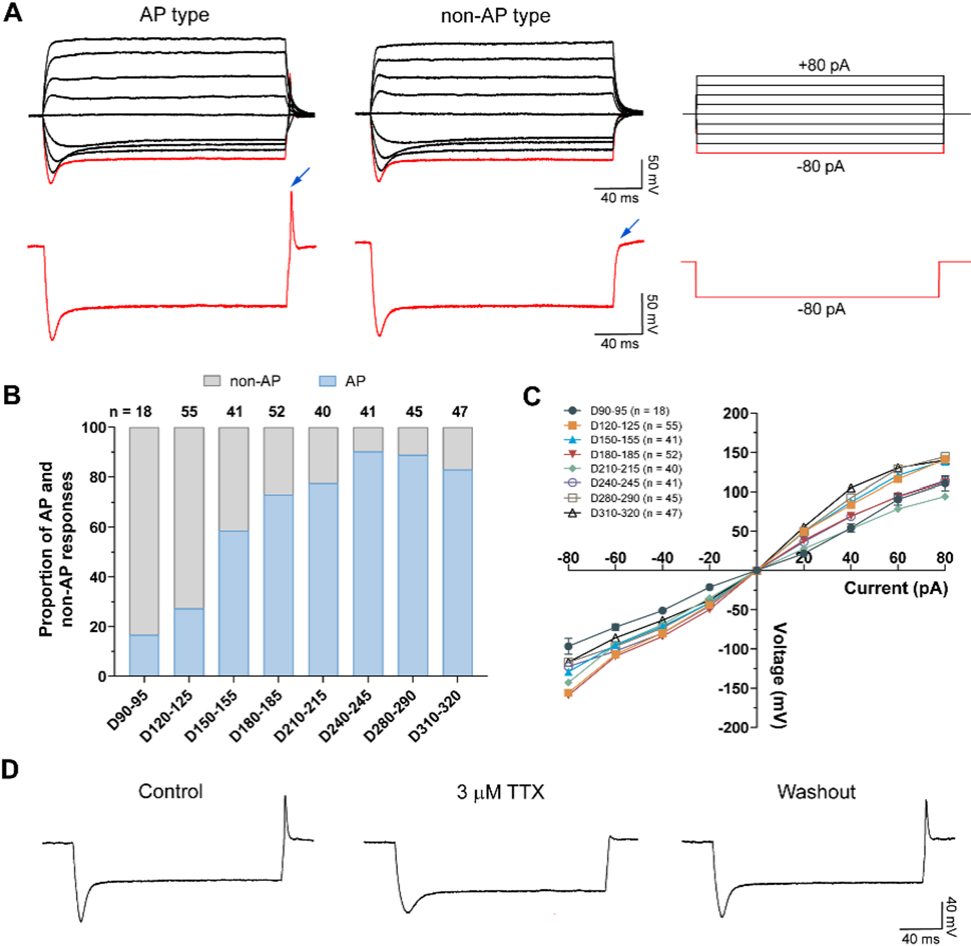
Voltage responses to hyperpolarizing and depolarizing current steps in RO photoreceptors. **A** Representative traces of two types of membrane potentials in response to current steps from − 80 pA to + 80 pA in 20-pA increments. Action potential type (AP type), action potentials of a RO photoreceptor occurred during the recovery phase of hyperpolarizing responses; non-action potential type (non-AP type), no action potentials occurred during the recovery phase of hyperpolarizing responses. Right traces, current stimulation waveform used to elicit membrane potentials. Arrows indicate the spike-like potentials during hyperpolarization recovery in the AP type cell and the absence of such response in the non-AP type cell. **B** Fraction of AP and non-AP type responses across different stages of RO differentiation (n = number of cells). The proportion of AP type response increased during RO maturation, peaking at D240–245. **C** Current-voltage relationship for RO photoreceptors at various different differentiation stages (n = number of cells), illustrating the membrane potential changes in response to various current injections. **D** Representative traces of voltage responses to − 80 pA current injection in a RO photoreceptor at D240 of differentiation before treatment (Control), after application of voltage-gated sodium channel blocker TTX (3 µM TTX), and following washout of TTX (Washout). The AP was blocked by 3 µM TTX and recovered after washout

Mammalian photoreceptors undergo hyperpolarization in response to a light stimulus and are generally considered to be non-spiking neurons [36–38]. However, several studies have revealed Na^+^-spike-like responses in human photoreceptors [10, 24, 39]. To evaluate action potential (AP) generation capacity during human photoreceptor maturation, voltage responses of RO photoreceptors to current steps (− 80 to + 80 pA) were recorded across differentiation stages (D90–320). We identified two distinct electrophysiological phenotypes: (1) AP type cells exhibiting spike-like potentials during hyperpolarization recovery, and (2) Non-AP type cells lacking regenerative responses (Fig. 5A). Quantitative analysis demonstrated progressive acquisition of AP competence, with the proportion of AP type photoreceptors increasing from 16.7% (3/18) at D90–95 to 58.5% (24/41) by D150–155, peaking at 90.2% (37/41) during D240–245 before declining to 83.0% (39/47) at D310–320 (Fig. 5B). This developmental trajectory confirms stage-dependent functional maturation, where AP generation capacity reaches optimal levels at D240. Membrane properties remained stable throughout differentiation, as evidenced by consistent current-voltage relationships across all stages (Fig. 5C).

To further confirm the role of Nav channels in the generation of APs, we applied 3 µM TTX. Voltage responses to 80 pA current injection were recorded before treatment (Control), after application of TTX (3 µM TTX), and following washout of TTX (Washout) (Fig. 5D). The APs were blocked by 3 µM TTX and recovered after washout, confirming the involvement of Nav channels in the generation of APs in RO photoreceptors.

### Electrophysiological characterization of inward rectification in RO photoreceptors

**Fig. 6:**
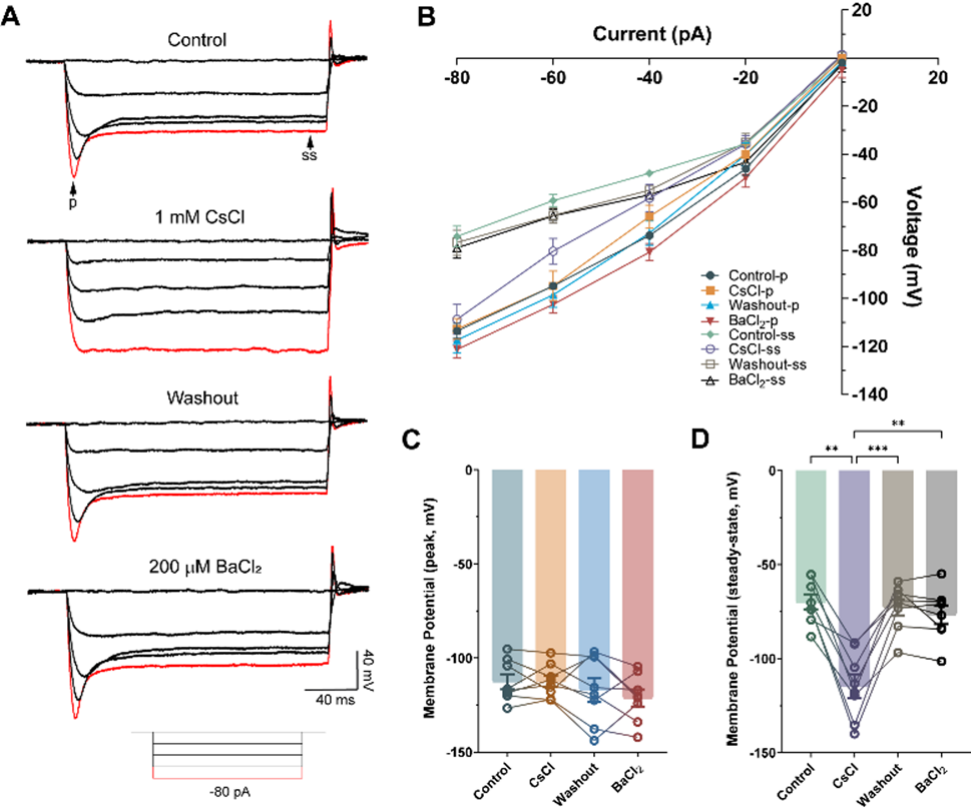
Characterization of inward rectification in RO photoreceptors. **A** Representative traces of membrane potential responses under control condition, application of 1 mM CsCl, washout and application of 200 µM BaCl_2_. Arrows indicate the peak (p) and steady state (ss) membrane potentials in response to hyperpolarizing current injections. Note that CsCl eliminated inward rectification, whereas BaCl_2_ had no effect on it. **B** Current-voltage relationship curve, summarizing the peak (p) and steady state (ss) membrane potentials across the different treatment groups (*n* = 10). **C** Peak membrane potential recorded during a − 80 pA current injection (*n* = 10). **D** Steady state membrane potential (ss) recorded under the same current injection conditions as in C (*n* = 10). Data were presented as mean ± SEM, and statistical significance was determined using one-way ANOVA with Tukey’s multiple comparisons test, *P < 0.05

Based on our initial observation of inward rectification in RO photoreceptors during membrane potential recordings (as shown in Fig. 5A), we sought to identify the ionic current responsible for this phenomenon. Therefore, we conducted current-clamp recordings during pharmacological profiling (Fig. 6A). Control conditions exhibited characteristic rectification with significant peak-to-steady-state potential differences. Bath application of 1 mM CsCl (3–5 min), a blocker of HCN channels, abolished rectification (steady-state: − 108.7 ± 6.3 mV vs. control − 74.2 ± 4.3 mV, **P < 0.01), while CsCl washout restored near-complete rectification (Fig. 6B and D). Conversely, 200 µM BaCl₂ (anomalous rectifier K channel blocker) caused no significant alteration (steady-state: − 79.0 ± 4.2 mV, Fig. 6B and D). Current–voltage relationships (Fig. 6B) confirmed these effects, with − 80 pA injections showing CsCl specifically eliminated the steady-state potential shift without affecting peak hyperpolarization (Fig. 6C). This pharmacological analysis establishes HCN-mediated I_*h*_ currents as the exclusive mechanism underlying inward rectification in mature RO photoreceptors.

## Discussion

The translational application of human pluripotent stem cell-derived ROs for disease modeling and cell replacement therapies is currently hindered by a lack of functionally validated quality control criteria. This study overcomes this limitation by establishing the first comprehensive electrophysiological maturation timeline for photoreceptors in long-term cultured ROs. Our systematic characterization reveals a defined developmental trajectory wherein membrane properties stabilize by days 120–125, indicating structural maturation. Notably, key functional hallmarks including hyperpolarization-activated (I_*h*_) currents and AP generation peak at day 240, achieving physiological levels comparable to mature primate and human photoreceptors. We establish day 240 as a definitive functional maturation timepoint, providing an essential physiological benchmark for standardizing RO quality control.

The development of ROs is a continuous and dynamic process, during which morphological, structural, and functional characteristics vary significantly across different developmental stages [40–42]. In the early stages of organoid development, cells primarily undergo proliferation and differentiation [15, 43], while later stages focus on the establishment of intercellular synaptic connections and refinement of phototransduction functionality [16, 30]. This stage-dependent heterogeneity results in distinct physiological states and properties at different time points. The inherent heterogeneity of ROs poses challenges for their clinical application, particularly in transplantation, where it directly impacts engraftment efficiency, success rates, and post-transplant functional stability [44]. Therefore, rigorous functional assessment and validation during in vitro differentiation are imperative before their clinical utilization. While traditional molecular detection methods, such as immunohistochemistry or qPCR for CRX/recoverin, can reveal temporal expression patterns of differentiation markers [11, 45], they fail to evaluate dynamic functional properties of ion channels. In this study, we established a quantitative electrophysiological framework to assess the functional maturation of photoreceptors in ROs using whole-cell patch-clamp recordings. Notably, functional maturation of HCN channels (Fig. 2G) was significantly delayed compared to their transcriptional upregulation (Fig. 1B), highlighting the superior sensitivity of electrophysiological characteristics in reflecting functional states.

HCN channels play a critical role in maintaining the functional integrity of photoreceptors by stabilizing membrane potential dynamics during light responses [26, 46–48]. Unlike their canonical role in generating rhythmic activity in cardiac or central neurons, retinal HCN channels act as voltage-dependent dampeners to prevent excessive membrane hyperpolarization caused by sustained light stimuli [39, 49, 50]. This regulation ensures precise temporal encoding of visual signals by limiting fluctuations in photoreceptor membrane potential [49]. Our electrophysiological data further elucidate their dynamic regulation during human photoreceptor development. The developmental trajectory of I_*h*_ currents in our hESC-derived ROs (hESC-ROs), which parallels the overall maturation timeline, is consistent with that reported in hiPSC-derived ROs (hiPSC-ROs) [10]. Notably, we detected differences in the I_*h*_ currents amplitude between hESC-ROs and hiPSC-ROs at equivalent differentiation stages. Furthermore, current kinetics resembling those of macaque photoreceptors were emerged in hESC-ROs as early as D120 and persisted through D320. This contrasts with the timing reported for hiPSC-ROs, where comparable kinetics typically observed at later stages (D200+) [10]. The distinct functional outputs observed here, particularly in I_*h*_ current amplitude and the onset of mature-like kinetics, may be attributed to a combination of factors. These potentially include inherent biological differences between stem cell sources (e.g., epigenetic priming [43]), as well as critical methodological variations in differentiation protocols. Accordingly, while both hESC-ROs and hiPSC-ROs exhibit similar transcriptional dynamics during retinal differentiation [51], our electrophysiological data suggest that the functional maturation pace and output may vary, and the underlying causes require further investigation under controlled, side-by-side comparisons. Beyond the variations among human stem cell-derived models, our electrophysiologically defined maturation timeline reveals a more profound divergence when placed in an interspecies context. In contrast to the rapid functional maturation of mouse photoreceptors, which occurs within weeks postnatally and exhibits stable I_*h*_ currents upon transplantation [52], our hESC-derived photoreceptors required an extended differentiation period exceeding 240 days to achieve full functional maturity. This stark disparity in developmental timescales highlights fundamental differences in the intrinsic pace of retinal development between primates and rodents [53]. Therefore, the extended timeline observed in our human ROs recapitulates a species-specific biological program of human retinogenesis. This comparison underscores the unique utility of long-term human RO models for faithfully capturing the extended kinetics of human photoreceptor maturation, which is essential for modeling age-related or late-onset retinal degenerative diseases.

Our study revealed that photoreceptors in ROs generate Na⁺-dependent APs during the recovery phase of hyperpolarizing responses, a finding that significantly challenges the traditional view of photoreceptors as “non-spiking neurons” [54]. While mammalian photoreceptors are generally thought to transmit signals solely through light-induced graded hyperpolarization [37, 55], accumulating evidence indicates that primate photoreceptors, particularly in humans, can generate Na⁺-dependent APs under specific conditions [24, 39, 56]. Our data demonstrate that ROs progressively acquire AP generating capacity during extended in vitro maturation, with the proportion of photoreceptors exhibiting APs increasing from 16.7% at D90–95 to 90.2% by D240–245 (Fig. 5B). This developmental trajectory mirrors the functional maturation of native human photoreceptors, where Na⁺ spikes are hypothesized to enhance neurotransmitter release kinetics during light offset, thereby improving temporal precision in visual signal transmission [24, 56]. TTX-sensitive nature of these APs (Fig. 5D) strongly implicates Nav channels in this phenomenon. In mammalian photoreceptors, Nav1.1 (*SCN1A*), Nav1.2 (*SCN2A*), and Nav1.6 (*SCN8A*) are predominantly expressed [57], with Nav1.6 reported as critical for the maturation of both rod and cone photoreceptors [58]. Its deficiency leads to severe defects in scotopic vision and may impair cone functionality, underscoring the importance of sodium channel subtypes in phototransduction. However, the specific Nav subtypes governing AP generation in photoreceptors remain poorly characterized, representing a key focus for future investigation. The discovery of APs in RO photoreceptors carries significant scientific implications. The developmental acquisition of AP-generating capacity provides a novel electrophysiological biomarker for assessing photoreceptor maturation in ROs, complementing existing morphological and molecular criteria.

The functional maturation timeline established in this study delivers transformative implications for retinal disease modeling and therapy development. By identifying D240 as a physiological benchmark, this work enables robust modeling of late-stage degenerative phenotypes, particularly the progressive attenuation of HCN channel function (e.g., I_*h*_ current decline > D240, Fig. 2G) that mirrors in vivo pathology in RP [59]. Crucially, HCN1 deficiency exacerbates photoreceptor degeneration in RP models, as evidenced by accelerated loss of retinal structure, pathological depolarization, and calcium-dependent calpain activation, which collectively drive signaling delays observed in advanced disease states. Furthermore, D240 defines an optimal therapeutic window for intervention studies. Targeted application of gene therapy vectors (e.g., AAV-mediated HCN1) or neuroprotective agents at this peak maturation phase permits rigorous evaluation of functional rescue on mature electrophysiological phenotypes. This approach overcomes limitations of immature models, where therapeutic efficacy may be misrepresented due to incomplete channel expression [59].

This study has several limitations. First, our current system does not permit definitive attribution of recorded electrophysiological signals to specific photoreceptor subtypes. Transcriptomic analysis of publicly available RNA-seq data (GEO: GSE136929) confirms that cone fate commitment occurs earlier than rod fate commitment [13], mirroring in vivo development [60, 61]. However, fate commitment does not equate to functional maturity. Key photopigment proteins (OPN1L/MW and RHO) become detectable by immunofluorescence at D150 (Fig. 1C), and hallmark currents such as I*ₕ* follow a protracted developmental trajectory peaking at D240. Given the overlapping timing and similar membrane properties of cones and rods, we cannot precisely distinguish their respective contributions to the functional maturation timeline established here. Future studies employing cell-type-specific reporters will be essential to resolve the distinct maturation trajectories of each subtype. Second, due to the high costs and technical challenges associated with long-term culture, especially maintaining organoid viability beyond D300, we were unable to systematically analyze the electrophysiological properties of ROs at later differentiation stages (e.g., D400+). Although characteristic features such as APs and HCN channel-mediated I_*h*_ currents were observed in a subset of D400 + ROs, the limited sample size precluded a detailed investigation of their electrophysiological properties. Comprehensive electrophysiological characterization of these advanced maturation stages is critical for evaluating the ultimate functional maturity of ROs, and future studies should focus on optimizing culture systems to reduce costs and extend experimental timelines. Third, the absence of light-response recordings limits our evaluation of ROs’ functional integrity. While attempts were made to record light-evoked responses, technical difficulties and low success rates hindered the acquisition of quantifiable data. This gap represents a common challenge in the field, and subsequent work will prioritize establishing standardized light-response detection platforms to address this limitation.

## Conclusion

This study establishes functional biomarkers and a definitive electrophysiological maturation timeline for photoreceptors in hESC-derived ROs. Our findings provide critical quality control standards for their in vitro differentiation and advance the utility of ROs for modeling retinal degenerative diseases and developing cell replacement therapies.

## Supplementary Information

Below is the link to the electronic supplementary material.

Supplementary Material 1. Figure S1. Identity validation and quality control of the H9-ESC-CRX-tdTomato photoreceptor reporter cell line. (A) Gel electrophoresis of PCR products from routine mycoplasma testing, showing a negative result (only the positive control band is visible). (B) Karyotype analysis of the H9-ESC-CRX-tdTomato cell line, confirming a normal diploid chromosomal profile. (C) Genotyping results for short tandem repeat (STR) loci and the Amelogenin locus in the submitted H9-ESC-CRX-tdTomato cell line, with allelic profiles matching the reference h9 cell bank. Figure S2. Identification of tdTomato-positive cells as photoreceptors at different stages in ROs. (A) Immunofluorescence analysis of CRX (yellow) and tdTomato (red) co-localization in ROs at different differentiation stages (D60, D90). (B) Immunofluorescence analysis of RCVRN (green) and tdTomato (red) co-localization at D320. CRX, cone-rod homeobox; RCVRN, recoverin. Nuclei were stained with DAPI (blue). Scale bar = 50 μm. Figure S3. Developmental maturation of Ih currents in RO-derived photoreceptors. (A-H) Representative whole-cell patch-clamp recordings of Ih currents traces at different differentiation stages: (A) D90, (B) D120, (C) D150, (D) D180, (E) D210, (F) D240, (G) D280, and (H) D310. Currents were elicited by hyperpolarizing voltage steps from -120 mV to -50 mV in 10-mV increments at holding potential of -50 mV. (I) Voltage waveform used to elicit Ih currents in A-H. Figure S4. Developmental maturation of Nav currents in RO-derived photoreceptors. (A-H) Representative whole-cell patch-clamp recordings of Nav currents traces at different differentiation stages: (A) D90, (B) D120, (C) D150, (D) D180, (E) D210, (F) D240, (G) D280, and (H) D310. Currents were elicited by hyperpolarizing voltage steps from -60 mV to +20 mV in 5-mV increments at holding potential of -90 mV. (I) Voltage waveform used to elicit Nav currents in A-H.

## Data Availability

The data that support the findings of this study are available within the article and its supplementary materials.

## Abbreviations

AMD: Age-related macular degeneration
AP: Action potential
CNG: Cyclic nucleotide-gated
CRX: Cone-rod homeobox
hESC: Human embryonic stem cell
HCN: Hyperpolarization-activated cyclic nucleotide-gated
I_h_: Hyperpolarization-activated current
Nav: Voltage-gated sodium
RCVRN: Recoverin
RO: Retinal organoid
RP: Retinitis pigmentosa
RMP: Resting membrane potential
TTX: Tetrodotoxin
