# Supplementary figures and images for: Functional development of photoreceptors in human retinal organoids
Source: Stem Cell Res Ther. 2026 Apr 30;17:223. doi: 10.1186/s13287-026-05027-z (PMC13277147; doi:10.1186/s13287-026-05027-z)

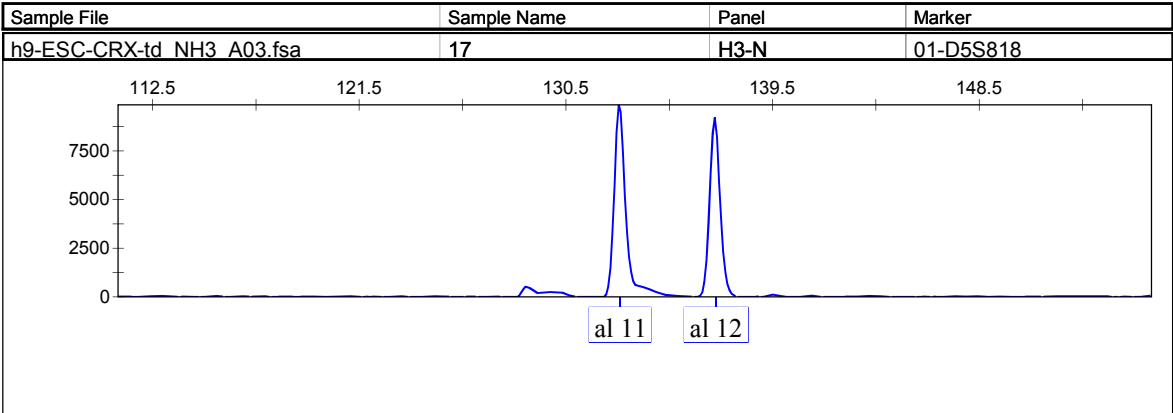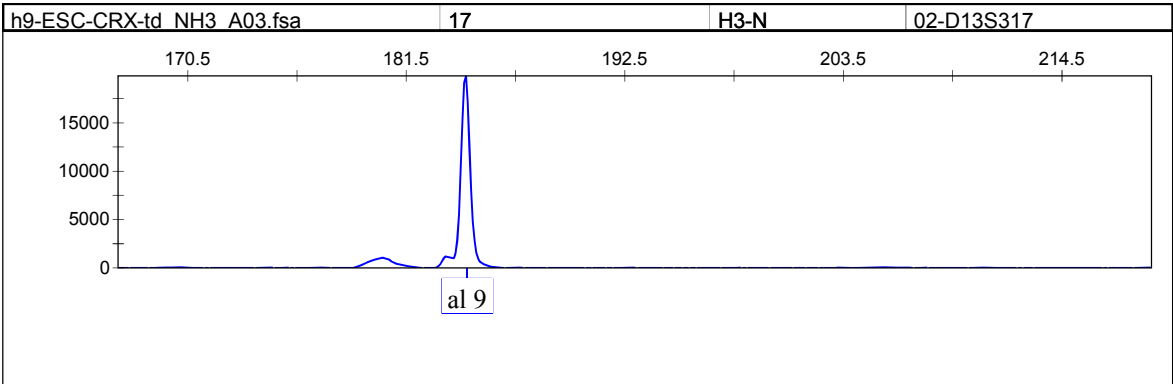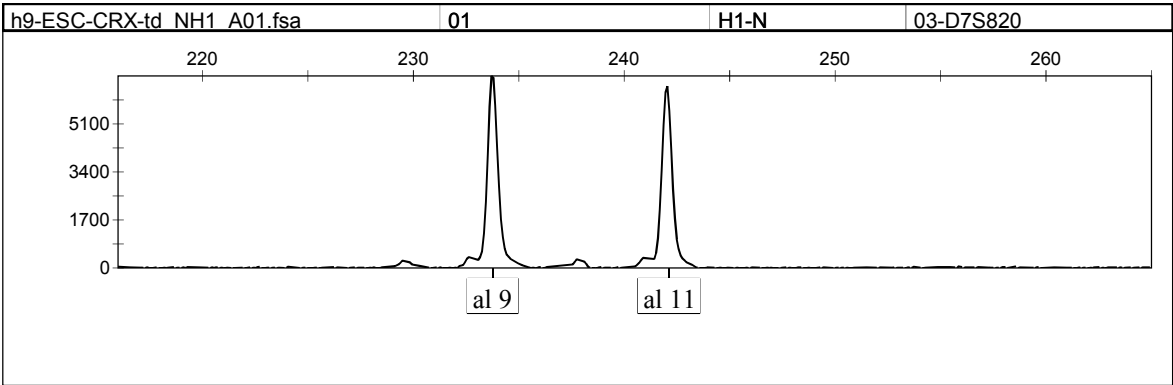

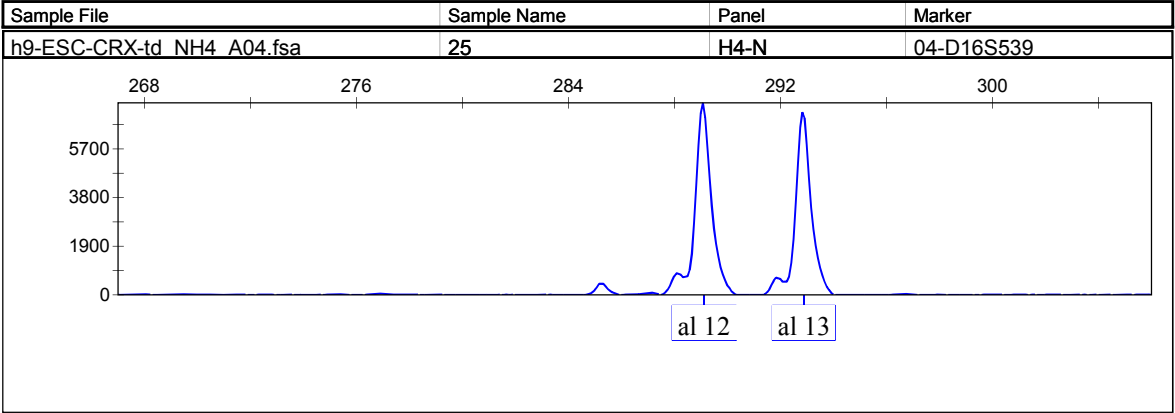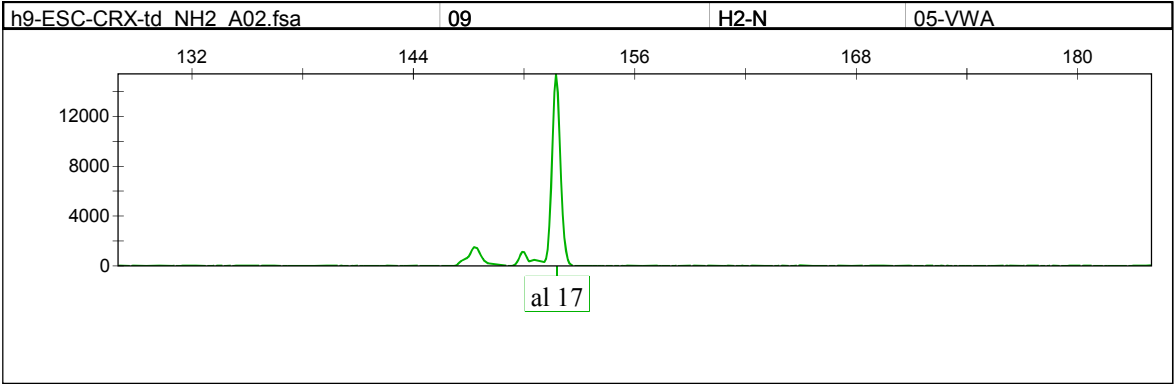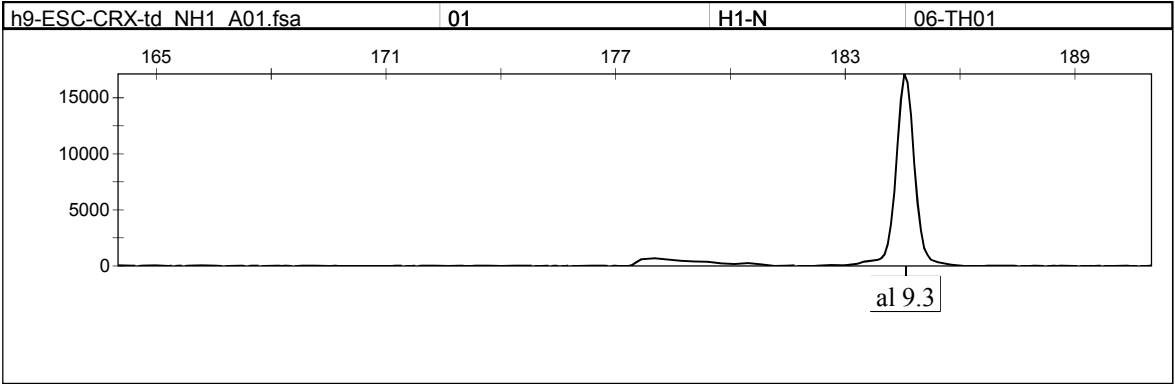

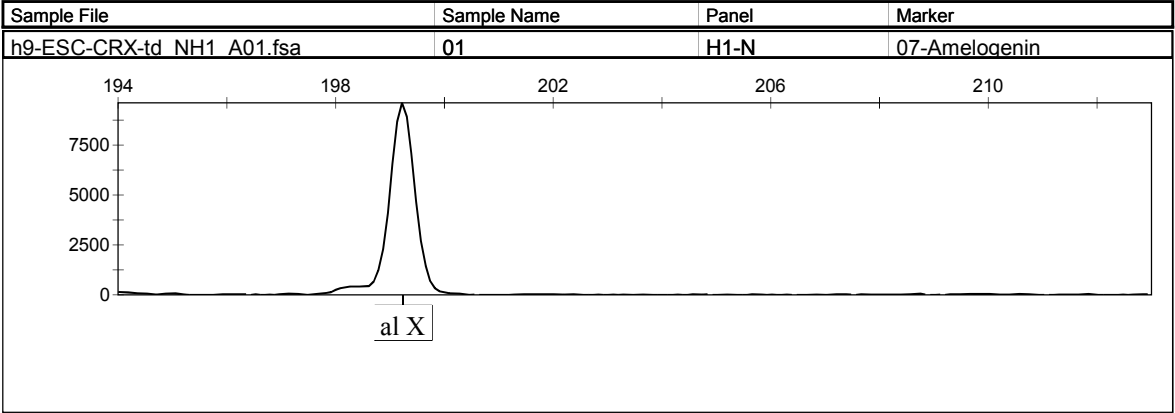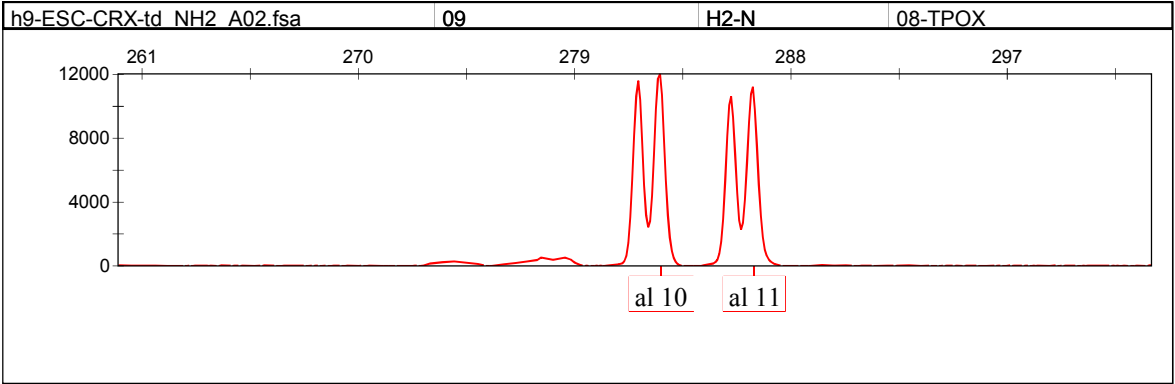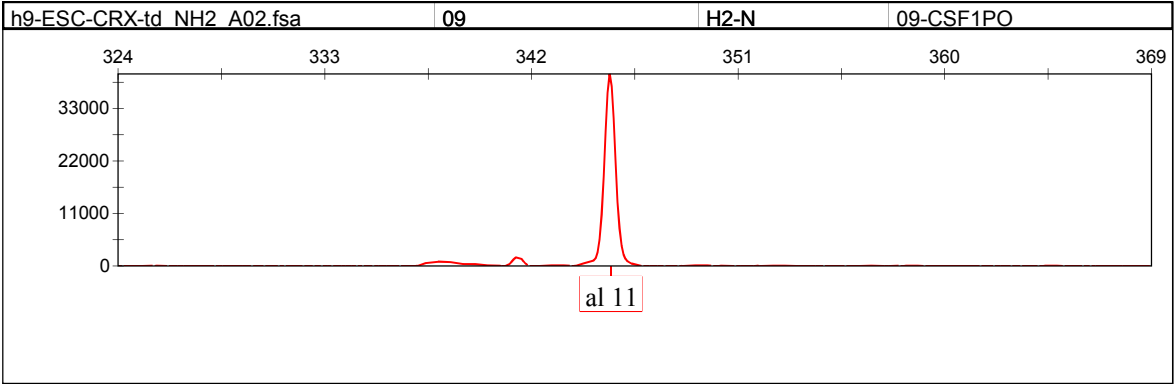

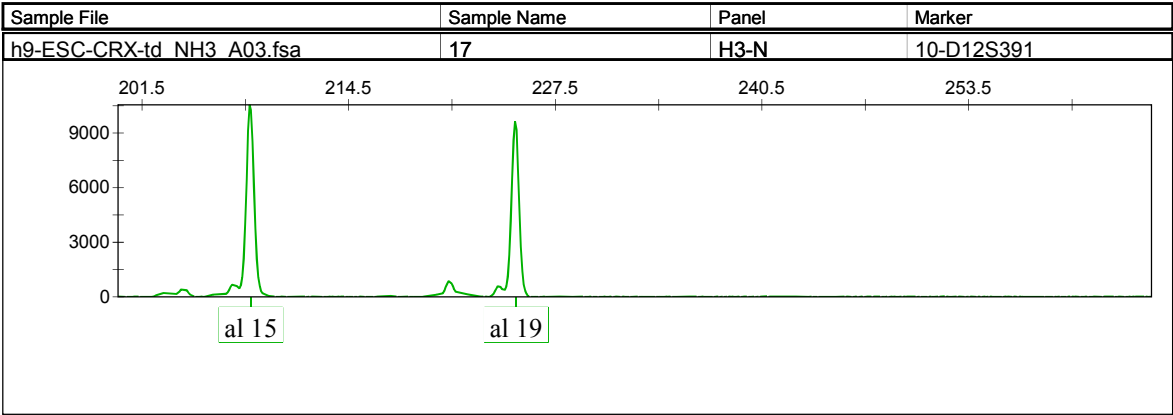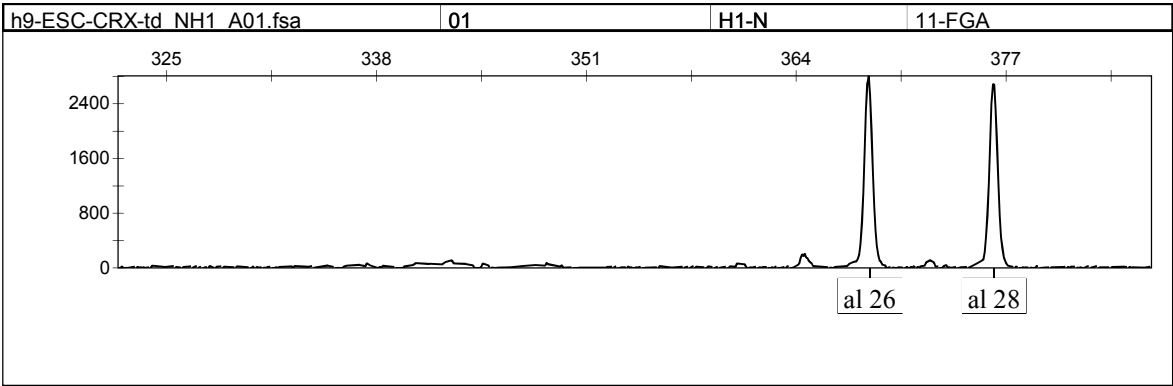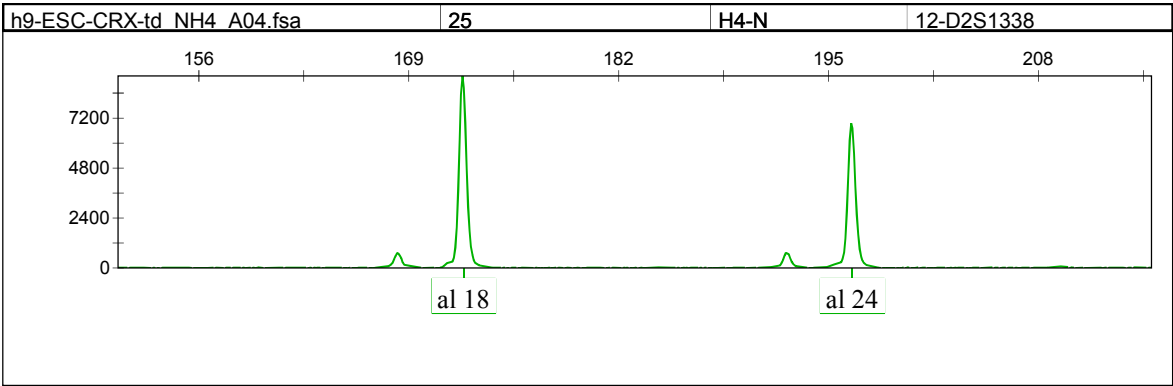

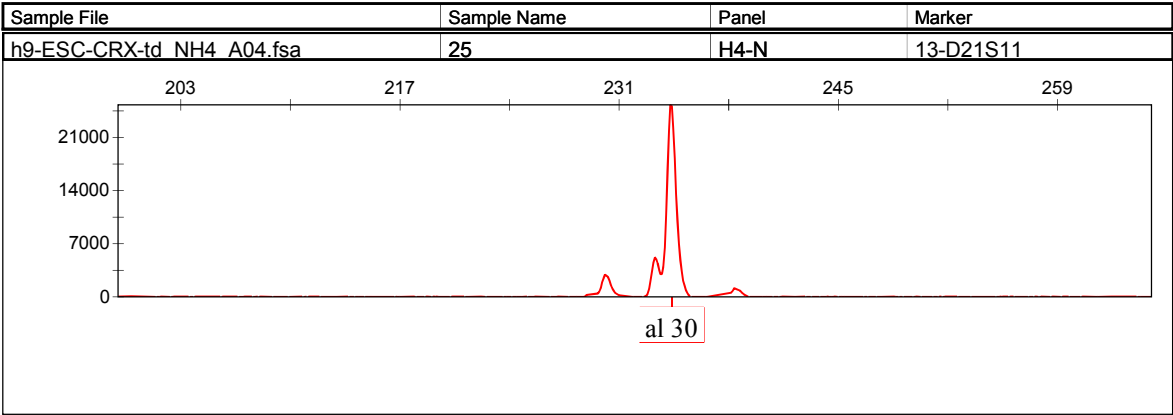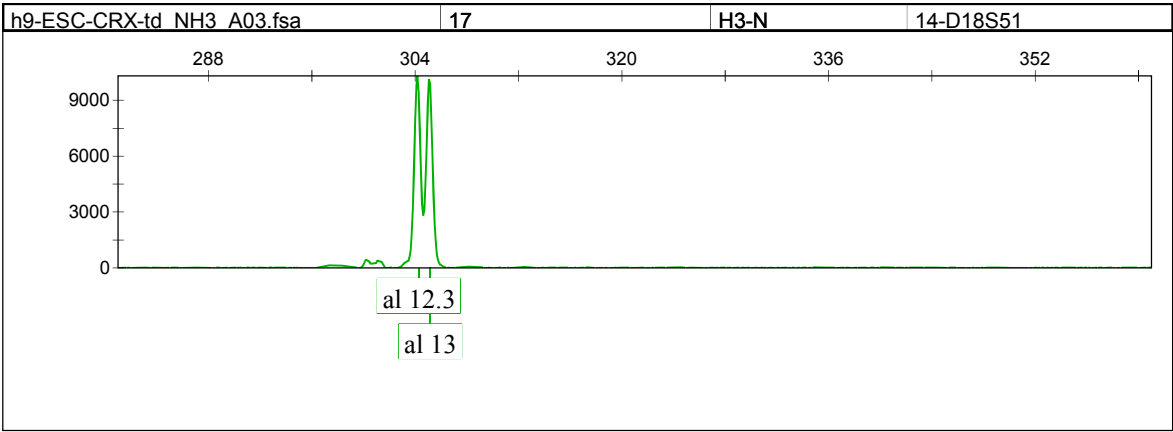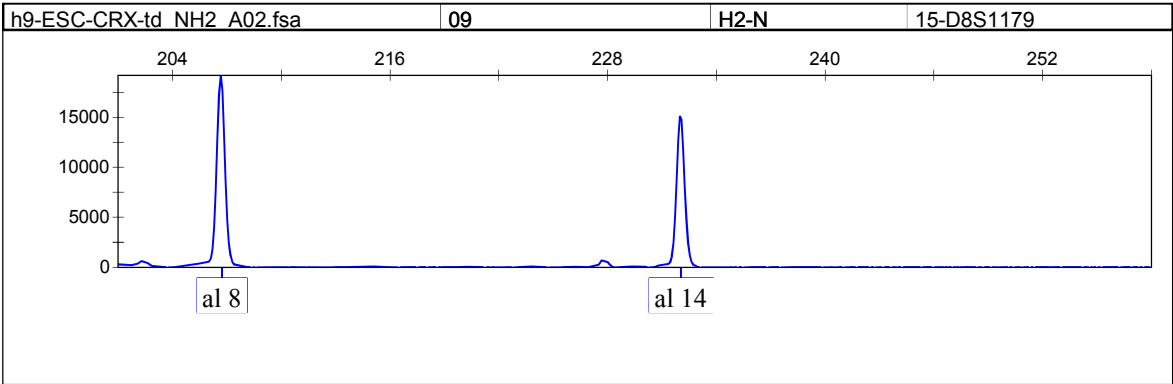

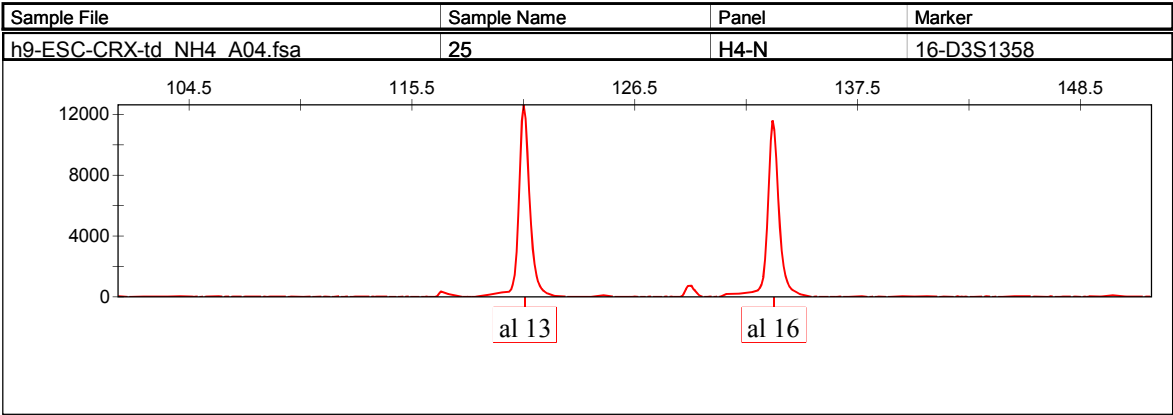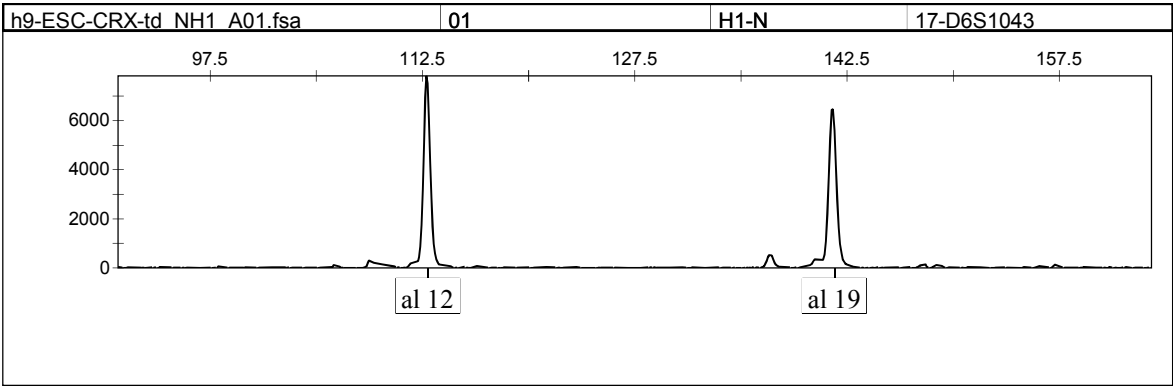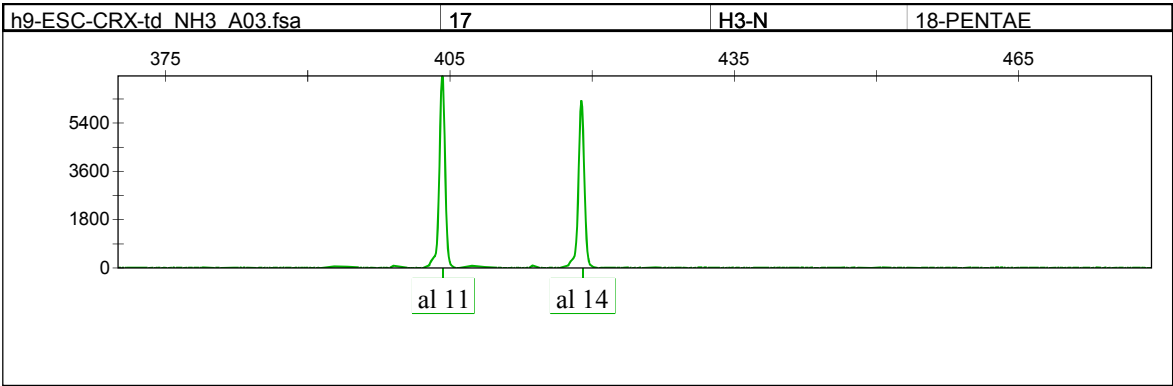

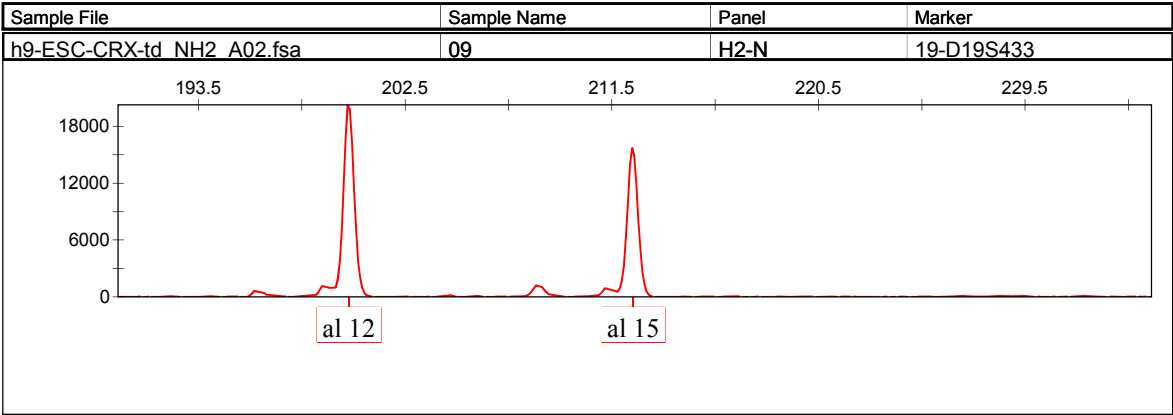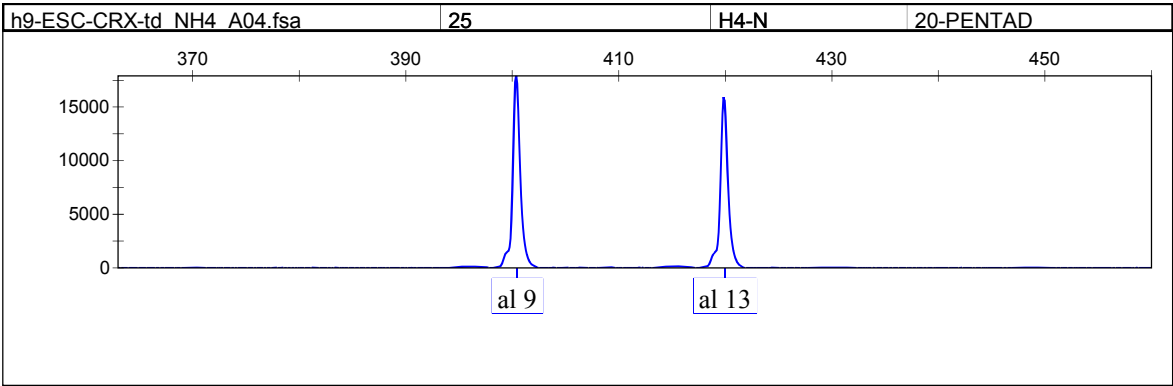

Supplement: Supplementary file 1 — Supplementary Material 1. Figure S1. Identity validation and quality control of the H9-ESC-CRX-tdTomato photoreceptor reporter cell line. (A) Gel electrophoresis of PCR products from routine mycoplasma testing, showing a negative result (only the positive control band is visible). (B) Karyotype analysis of the H9-ESC-CRX-tdTomato cell line, confirming a normal diploid chromosomal profile. (C) Genotyping results for short tandem repeat (STR) loci and the Amelogenin locus in the submitted H9-ESC-CRX-tdTomato cell line, with allelic profiles matching the reference h9 cell bank. Figure S2. Identification of tdTomato-positive cells as photoreceptors at different stages in ROs. (A) Immunofluorescence analysis of CRX (yellow) and tdTomato (red) co-localization in ROs at different differentiation stages (D60, D90). (B) Immunofluorescence analysis of RCVRN (green) and tdTomato (red) co-localization at D320. CRX, cone-rod homeobox; RCVRN, recoverin. Nuclei were stained with DAPI (blue). Scale bar = 50 μm. Figure S3. Developmental maturation of Ih currents in RO-derived photoreceptors. (A-H) Representative whole-cell patch-clamp recordings of Ih currents traces at different differentiation stages: (A) D90, (B) D120, (C) D150, (D) D180, (E) D210, (F) D240, (G) D280, and (H) D310. Currents were elicited by hyperpolarizing voltage steps from -120 mV to -50 mV in 10-mV increments at holding potential of -50 mV. (I) Voltage waveform used to elicit Ih currents in A-H. Figure S4. Developmental maturation of Nav currents in RO-derived photoreceptors. (A-H) Representative whole-cell patch-clamp recordings of Nav currents traces at different differentiation stages: (A) D90, (B) D120, (C) D150, (D) D180, (E) D210, (F) D240, (G) D280, and (H) D310. Currents were elicited by hyperpolarizing voltage steps from -60 mV to +20 mV in 5-mV increments at holding potential of -90 mV. (I) Voltage waveform used to elicit Nav currents in A-H. [file 13287_2026_5027_MOESM1_ESM.pdf]
